# Prenatal consequences of GALT deficiency in a rat model of classic galactosemia

**DOI:** 10.1371/journal.pgen.1012276

**Published:** 2026-09-21

**Authors:** Shauna A. Rasmussen, Catherine M. Lemons, Madelyn M. Seemiller, Sabrina R. Dallas, Milan Cambon Ledesma, Yuhan Wu, Lauren E. Anshen, Ashlyn Farnham, Olivia S. Garrett, Judith L. Fridovich-Keil

**Affiliations:** 1 Department of Human Genetics, Emory University School of Medicine, Atlanta, Georgia, United States of America; 2 Emory College of Arts and Sciences, Emory University, Atlanta, Georgia, United States of America; University of Pennsylvania, UNITED STATES OF AMERICA

## Abstract

Classic galactosemia (CG) is a potentially lethal disease that results from profound deficiency of galactose-1-P uridylyltransferase (GALT). Prior reports document elevated galactose metabolites beginning *in utero* in CG, raising concern that long-term complications in CG might also trace their origins to the prenatal period. However, studies document that elevated galactose metabolites in the postnatal period do not predict later developmental complications, challenging the connection. Here, we explored the relationship between elevated galactose metabolites *in utero* and fetal outcome using a GALT-null rat model of CG. To modify galactose metabolites *in utero*, we arranged rat crosses to produce GALT-null pups developing within either a GALT-null or heterozygous dam, and with or without heterozygous siblings. We reasoned that GALT activity present *outside* the fetus, but within the maternal-fetal compartment, might limit metabolic abnormalities in a developing GALT-null fetus -- and it did. Specifically, while galactose metabolites in GALT-null fetal pups from all crosses remained elevated over controls, the levels varied. When the dam and some siblings were heterozygous, amniotic fluid galactitol from GALT-null fetal pups at GD21 was comparable to the levels reported from second trimester human CG amniotic fluid. When the dam was GALT-null, but some siblings were heterozygous, the median galactitol level in amniotic fluid was 2.45-fold higher. When the dam and all siblings were GALT-null, the median amniotic fluid galactitol level was 5.67-fold higher. GALT-null fetal pups also showed significant growth delay *in utero*, but only when the dam and all siblings were GALT-null. That growth of GALT-null fetal pups was fully rescued by gestation within a heterozygous dam despite the persistence of moderately elevated galactose metabolites raises the possibility that babies with CG developing within a carrier mother might also be at least partially protected *in utero* from the adverse consequences of their GALT-deficiency.

## Author summary

Metabolic abnormalities in classic galactosemia can be detected *in utero*, raising concern that long-term complications might also trace their origins to the prenatal period. Here, we addressed the relationship between prenatal metabolic abnormality and phenotypic consequence using a GALT-null rat model for classic galactosemia. Our results demonstrate that partial metabolic rescue conferred by developing within a heterozygous dam fully rescues the growth delay otherwise seen in GALT-null fetal pups. This finding challenges the connection between moderate prenatal metabolic abnormality and complications in CG.

## Introduction

Classic galactosemia (CG) is a rare autosomal recessive disorder that results from profound deficiency of galactose-1-P uridylyltransferase [1], the middle enzyme in the Leloir pathway of galactose metabolism [2] (Fig 1). Deficiency of other Leloir pathway enzymes results in other forms of galactosemia [3,4]. Most babies with CG are born apparently healthy [1], but with moderately elevated galactose metabolites in both cord [5] and peripheral blood [1]. A small number of studies also document moderately elevated galactose metabolites in amniotic fluid and affected human fetal tissues as early as the first trimester [6,7].

**Fig 1:**
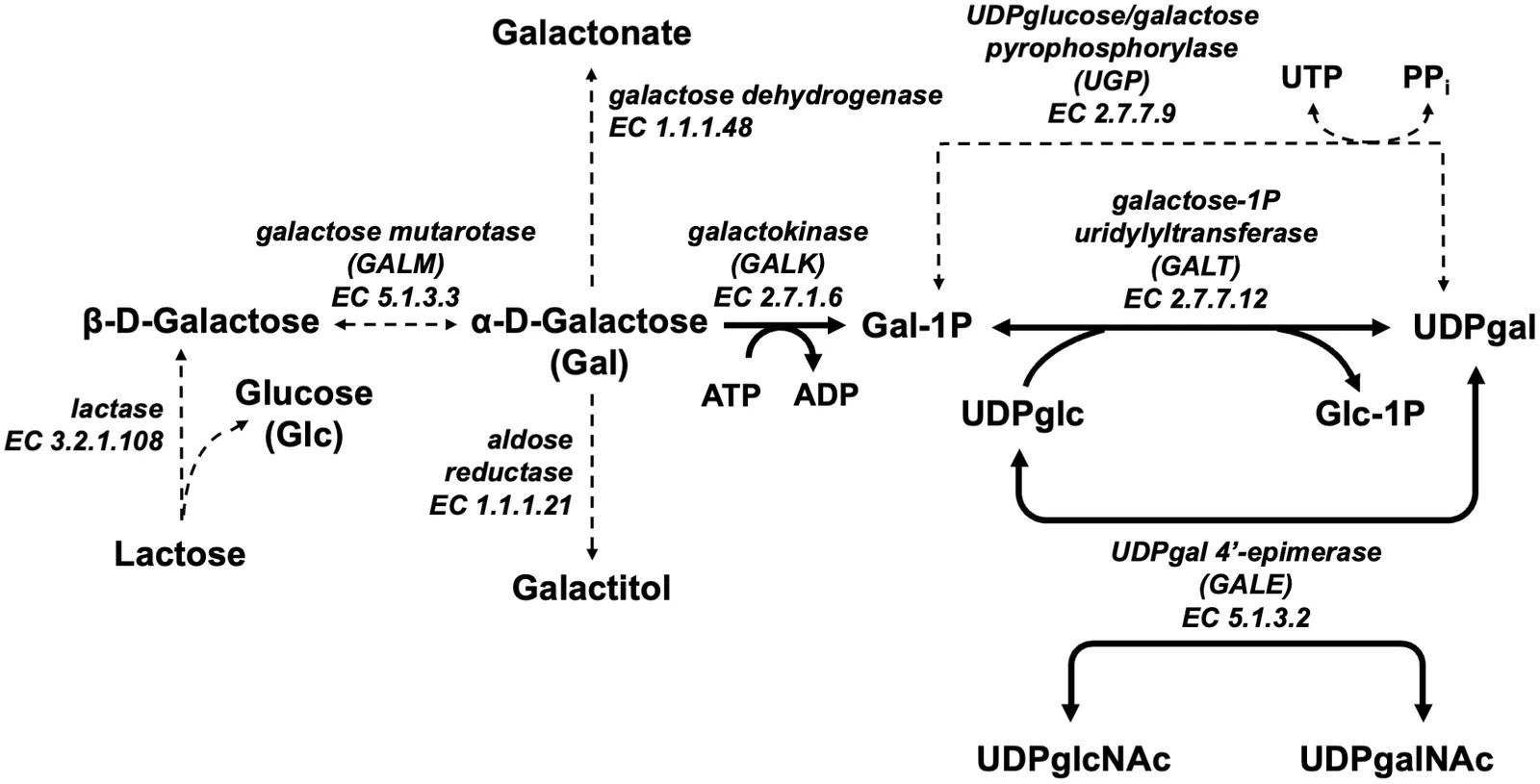
Pathways of galactose metabolism in human and rat. Metabolites are presented in standard font; enzymes with EC numbers are presented in italic font. Reactions catalyzed by enzymes of the conserved Leloir pathway, starting with GALK, are represented with solid arrows. Reactions catalyzed by other enzymes are represented with dashed arrows.

Shortly after birth, if a baby with CG consumes breast milk or a dairy milk-based formula, both of which contain abundant galactose in the form of lactose [8], blood and tissue galactose metabolites including galactose, galactitol, and galactose-1-phosphate (gal-1P) (Fig 1) spike dramatically [1]. As these metabolites rise, most affected infants also experience a rapid progression of acute clinical symptoms that can lead to neonatal death if the baby is not switched quickly to a low-galactose diet [1]. Following dietary intervention, blood and urine galactose metabolites return to a baseline that is slightly above the reference range, and the acute symptoms of disease resolve or are prevented. However, by elementary school many children with CG have been diagnosed with developmental or other long-term complications [9].

The relationship between elevated galactose metabolites and long-term complications in CG remains unclear and has been a point of contention for many years [10]. Because elevated galactose metabolites associate with acute disease in CG, many authors have assumed they must also underlie the long-term complications of CG. For example, some authors have proposed that gal-1P must be the toxic metabolite [11,12], while others have proposed galactitol [13], and yet others have suggested the pathophysiology of disease in CG may involve a combination of metabolites [14]. Contradicting these arguments, however, multiple studies have tested whether elevated galactose metabolites in newborns or older children associate with long-term complications in CG [10], and most have found they do not [9,15–17]. Further, that children with Duarte variant galactosemia (DG), who exhibit about 25% the normal level of GALT activity [18] and also experience moderately elevated galactose metabolites as babies if they drink milk, yet show no increased risk for developmental complications over controls [19], further challenges the relationship between elevated galactose metabolites and adverse long-term developmental outcomes in CG.

Here we report studies probing the relationship between elevated galactose metabolites *in utero* and adverse phenotypic consequences using our GALT-null rat model for CG [20]. These rats carry a frame-shift mutation in *GALT* that disrupts the enzyme active site and leads to a premature stop codon [20]. Prior studies of rats homozygous for this mutation have confirmed a complete absence of detectable GALT activity in all tissues tested, and accumulation of the same galactose metabolites reported in CG patients. Prior studies have also documented long-term complications in these GALT-null rats that are reminiscent of patient outcomes [20].

Previously, we noted that while GALT-null rat pups are born in the expected Mendelian ratios from cross types involving 1 or both heterozygous parents, ruling out increased fetal demise, the survival rate of GALT-null pups after birth is strikingly different between these cross types [20]. Specifically, GALT-null pups from GALT-null (-/-) female x GALT-null (-/-) male crosses show significantly lower survival in the first days after birth than do GALT-null pups from crosses where 1 or both parents were heterozygous [20]. This difference implicates the existence of modifying interactions either before birth in the maternal-fetal compartment, or after birth in the nest.

Distinguishing between these possibilities can be difficult because of the interdependence of rat maternal behavior and fetal health. For example, a rat mother may refuse to nurse a pup she deems unhealthy, dooming it to early demise. If nursed, a GALT-null rat pup may also struggle to fully metabolize its mother’s milk, which contains galactose [8], albeit far less than is found in human or cow’s milk [8]. Finally, it is possible that rat maternal behavior toward an entire litter may differ when all the pups are GALT-null versus when the litter is mixed; for example, due to a subtle difference in combined scent, or vocalizations, or some other cryptic factor.

Here, we used our GALT-null rats to test the possibility of metabolic and potentially also phenotypic rescue *in utero.* Specifically, we asked whether partial metabolic rescue of a GALT-null fetal pup *in utero* by GALT activity in the dam or siblings might also rescue an adverse phenotypic outcome – fetal growth delay. We did observe rescue. Considering that infants with CG are typically born to heterozygous (carrier) mothers, and at normal birth weight [9], our results may have implications for how we understand the timing and origins of long-term adverse outcomes in CG.

## Results

### Partial rescue of maternal and fetal galactose metabolite accumulation

#### Amniotic fluid.

As a first test of possible metabolic rescue *in utero* between GALT-null fetal pups and their mother or siblings, we compared the levels of galactose and galactitol in amniotic fluid samples collected at GD21, approximately 2–3 days before birth. Samples were harvested from euthanized dams representing each of 4 different types of crosses: (i) GALT-null (-/-) female x GALT-null (-/-) male, (ii) GALT-null (-/-) female x heterozygous (+/-) male, (iii) heterozygous (+/-) female x GALT-null (-/-) male, and (iv) wild-type (+/+) female x wild-type (+/+) male (Fig 2**, panels A and B**).

**Fig 2:**
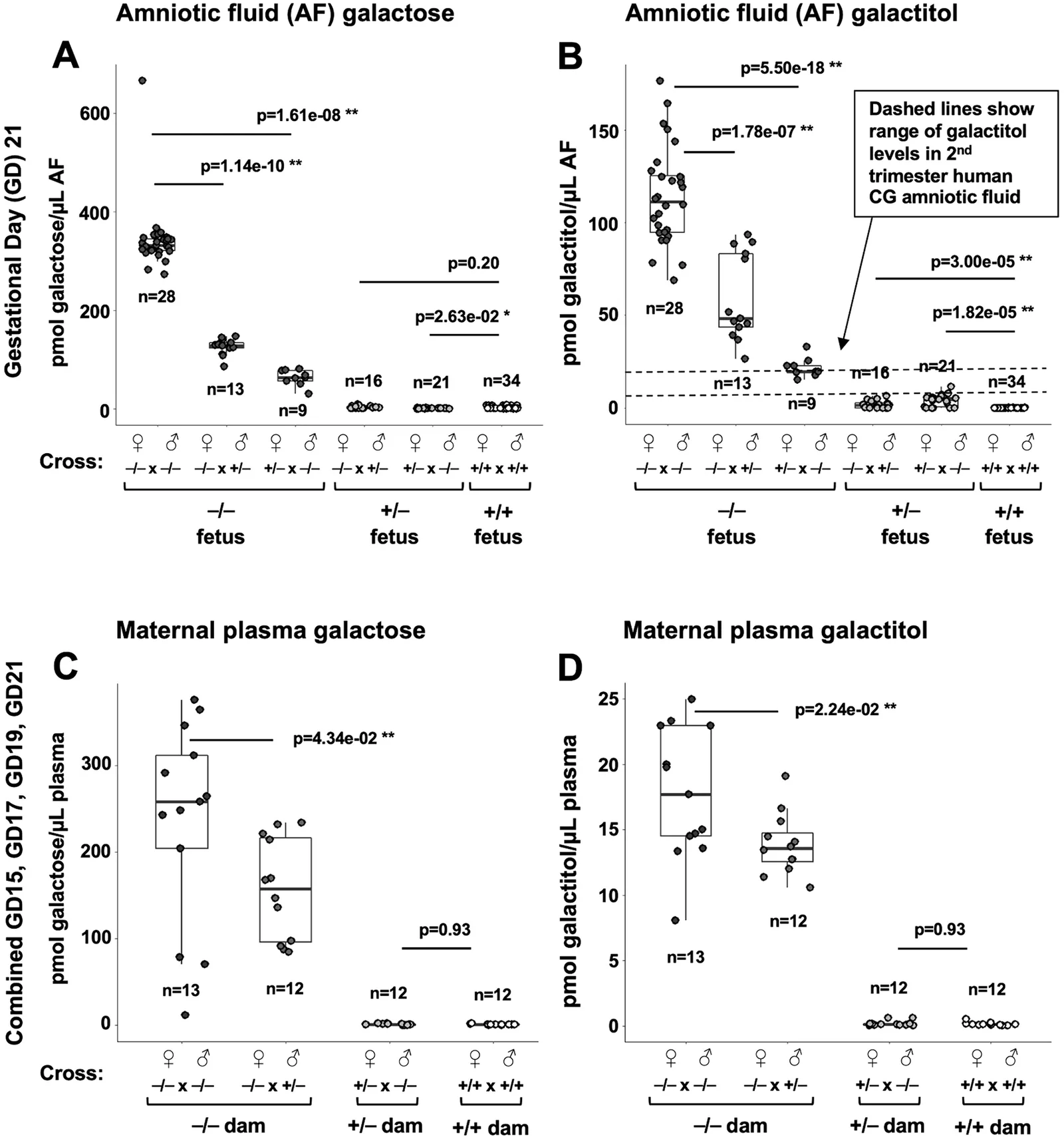
Partial metabolic rescue in rat amniotic fluid at GD21 and maternal plasma at GD15, 17, 19, and 21 (combined). **(A)** Galactose levels in amniotic fluid collected at GD21 from GALT-null, heterozygous, and wild-type fetal pups from the indicated cross types. **(B)** Galactitol levels in amniotic fluid collected at GD21 from GALT-null, heterozygous, and wild-type fetal pups from the indicated cross types. Dashed lines show the range of galactitol levels reported from 2^nd^ trimester human CG amniotic fluid [21]. **(C)** Galactose levels in maternal plasma collected at GD15, 17, 19, and 21 (combined) from GALT-null, heterozygous, and wild-type dams from the indicated cross types. **(D)** Galactitol levels in maternal plasma collected at GD15, 17, 19, and 21 (combined) from GALT-null, heterozygous, and wild-type dams from the indicated cross types. * = nominally significant, ** = significant after Bonferroni multiple test correction (if needed).

Both galactose and galactitol levels in amniotic fluid were abnormal in GALT-null fetal pups from all cross types, but the degree of abnormality varied markedly. Specifically, both metabolites were moderately elevated when both the dam and some siblings were heterozygotes (Fig 2**, panels A and B**), and these galactitol levels (median 19.64 pmol/µL; range 15.31 to 32.85 pmol/µL) overlapped with the galactitol levels reported from second trimester amniotic fluid collected from heterozygous women carrying a CG fetus (range 5.9 to 18.7 µmol/L [21,22]) (Fig 2**, panel B**, dashed lines). When the dam was GALT-null, but some siblings were heterozygotes, the median galactitol level in amniotic fluid was 2.45-fold higher (median 48.17 pmol/µL; range 26.54 to 93.61 pmol/µL). When both the dam and all siblings were GALT-null, the median galactitol was 5.67-fold higher (median 111.31 pmol/µL; range 68.79 to 176.37 pmol/µL; Fig 2**, panels A and B**).

#### Maternal plasma.

The galactose and galactitol levels in plasma samples from GALT-null dams also showed evidence of rescue when approximately half of the pups they carried were heterozygotes (Fig 2**, panels C and D;** S1 Fig). As in fetal pup amniotic fluid, the plasma metabolic correction observed in these GALT-null dams was significant, although the metabolite levels tested were not fully normalized.

#### Fetal liver and brain.

Metabolites in fetal liver and brain also showed evidence of rescue at GD21, but with some exceptions. For example, we saw strong rescue of galactose, galactitol, and galactose-1-phosphate (gal-1P) levels in fetal brain (Fig 3 **panels D, E, and F**), but only limited or inconsistent rescue in fetal liver (Fig 3 **panels A, B and C**). As in other sample types, heterozygous and wild-type fetal pups showed near-zero levels of all 3 metabolites in both liver and brain (Fig 3**, all panels**).

**Fig 3:**
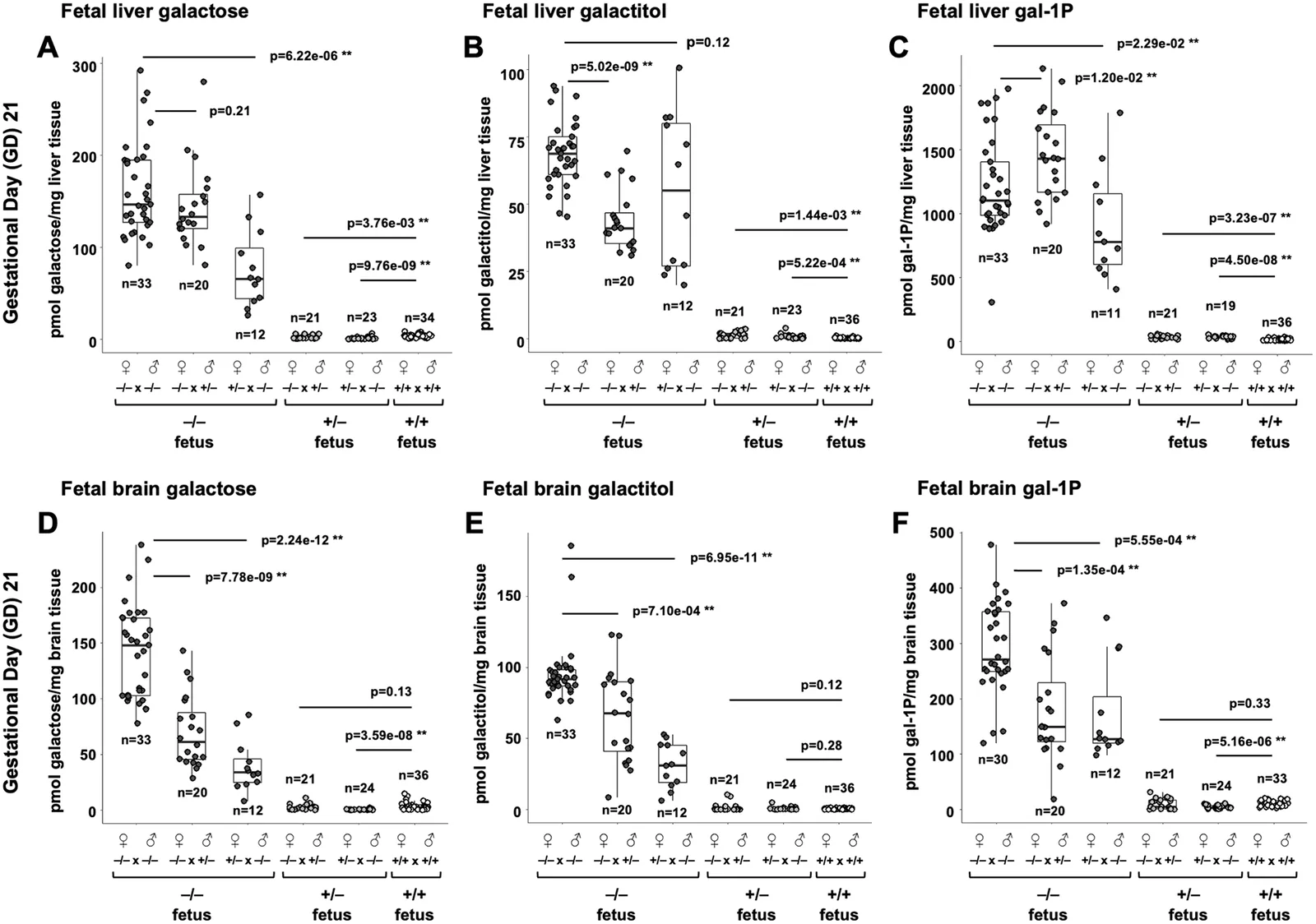
Partial metabolic rescue in rat fetal liver and brain at GD21. **(A)** Galactose levels in liver collected at GD21 from GALT-null, heterozygous, and wild-type fetal pups from the indicated cross types. **(B)** Galactitol levels in liver collected at GD21 from GALT-null, heterozygous, and wild-type fetal pups from the indicated cross types. **(C)** Gal-1P levels in liver collected at GD21 from GALT-null, heterozygous, and wild-type fetal pups from the indicated cross types. **(D)** Galactose levels in brain collected at GD21 from GALT-null, heterozygous, and wild-type fetal pups from the indicated cross types. **(E)** Galactitol levels in brain collected at GD21 from GALT-null, heterozygous, and wild-type fetal pups from the indicated cross types. **(F)** Gal-1P levels in brain collected at GD21 from GALT-null, heterozygous, and wild-type fetal pups from the indicated cross types. * = nominally significant, ** = significant after Bonferroni multiple test correction (if needed).

#### Metabolites at earlier gestational ages.

At earlier gestational ages (GD15, GD17, and GD19), we saw similar patterns. Specifically, GALT-null pups developing within a heterozygous dam demonstrated significant, albeit incomplete, rescue of elevated galactose metabolites in amniotic fluid (S2 Fig), and mixed impact in fetal liver (S3 Fig), and brain (S4 Fig).

We also tested the relative distribution of galactose metabolites in amniotic fluid, liver, and brain at GD17, GD19, and GD21 in GALT-null fetal pups from GALT-null (-/-) x GALT-null (-/-) crosses (S5 Fig). We excluded GD15 from this analysis due to the very low absolute levels of metabolites detected. In amniotic fluid, galactose predominated at all time points; in fetal liver, gal-1P predominated and galactitol was least abundant. In fetal brain, gal-1P again predominated and galactitol was almost as abundant as galactose. While these general patterns held at all 3 time points, we did see significant differences in the distribution of metabolites between the gestational ages tested. Further comparisons of metabolite distribution between liver and brain at each gestational age also yielded highly significant results (most p-values < 3e-04).

#### Testing possible fetal nearest neighbor effects on metabolites *in utero.*

Next, we asked whether the degree of metabolic correction evident in a GALT-null fetus gestating in the presence of heterozygous siblings within a GALT-null dam was influenced by whether it had 0, 1, or 2 heterozygous immediate neighbors. If we did see “immediate neighbor effects,” that might imply the mechanism of rescue was local, within the uterus. If we did not see immediate neighbor effects, that might imply the mechanism of rescue was more global and perhaps secondary to metabolic rescue of the maternal blood. The results (S6 Fig) showed limited, if any, immediate neighbor effects.

### Rescue of fetal growth delay at GD21

As an accessible measure of fetal outcome *in utero*, we recorded fetal mass. Previously, we have demonstrated that GALT-null rats show significant growth delay after birth relative to their heterozygous and wild-type counterparts [20,23], mimicking the postnatal growth delay seen in young CG patients (e.g., [9,16,17,24–26]). However, whether GALT-null rats also demonstrate growth delay before birth has not previously been reported.

At GD15, we saw no evidence of growth delay among GALT-null (-/-) fetal pups regardless of cross type (Fig 4**, panel A**), but at GD21 (Fig 4**, panel B**), we did. Specifically, GALT-null fetal pups were significantly smaller than their heterozygous (+/-) or wild-type (+/+) counterparts, but only if the dam was also GALT-null. GALT-null fetal pups developing within a heterozygous dam showed mass at GD21 indistinguishable from that of their heterozygous and wild-type counterparts (Fig 4**, panel B**).

**Fig 4:**
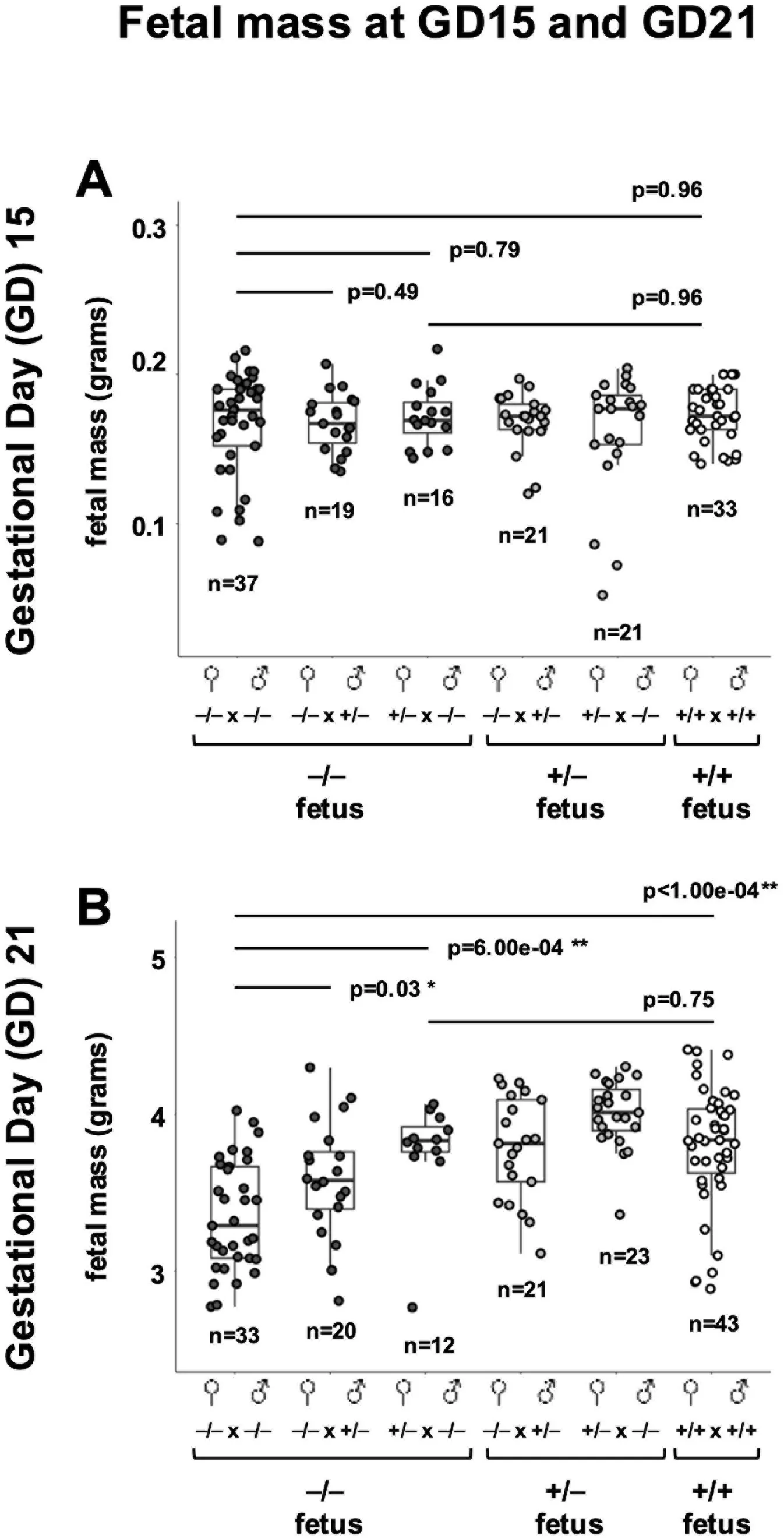
Correction of growth delay in GALT-null fetal pups. **(A)** Mass of GALT-null, heterozygous, and wild-type fetal pups from the indicated cross types at GD15. **(B)** Mass of GALT-null, heterozygous, and wild-type fetal pups from the indicated cross types at GD21. * = nominally significant, ** = significant after Bonferroni multiple test correction (if needed).

### Liver and brain GALT activities in pre- and post-natal rats

To ask whether the partial metabolic rescue of GALT-deficiency observed before birth in the maternal-fetal compartment might reflect a transfer of GALT-positive cells from the dam, or among siblings *in utero*, we quantified GALT activity in liver and brain from GALT-null fetal pups at GD21 from all 3 cross types. As a control, we also measured GALT activity in liver and brain from heterozygous and wild-type fetal pups. We saw no detectable GALT activity in samples of liver or brain from GALT-null fetal pups regardless of cross type. As expected, we did see intermediate activity in tissues from heterozygous fetal pups, and high activity in tissues from wild-type fetal pups (Fig 5**, panels A and B**).

**Fig 5:**
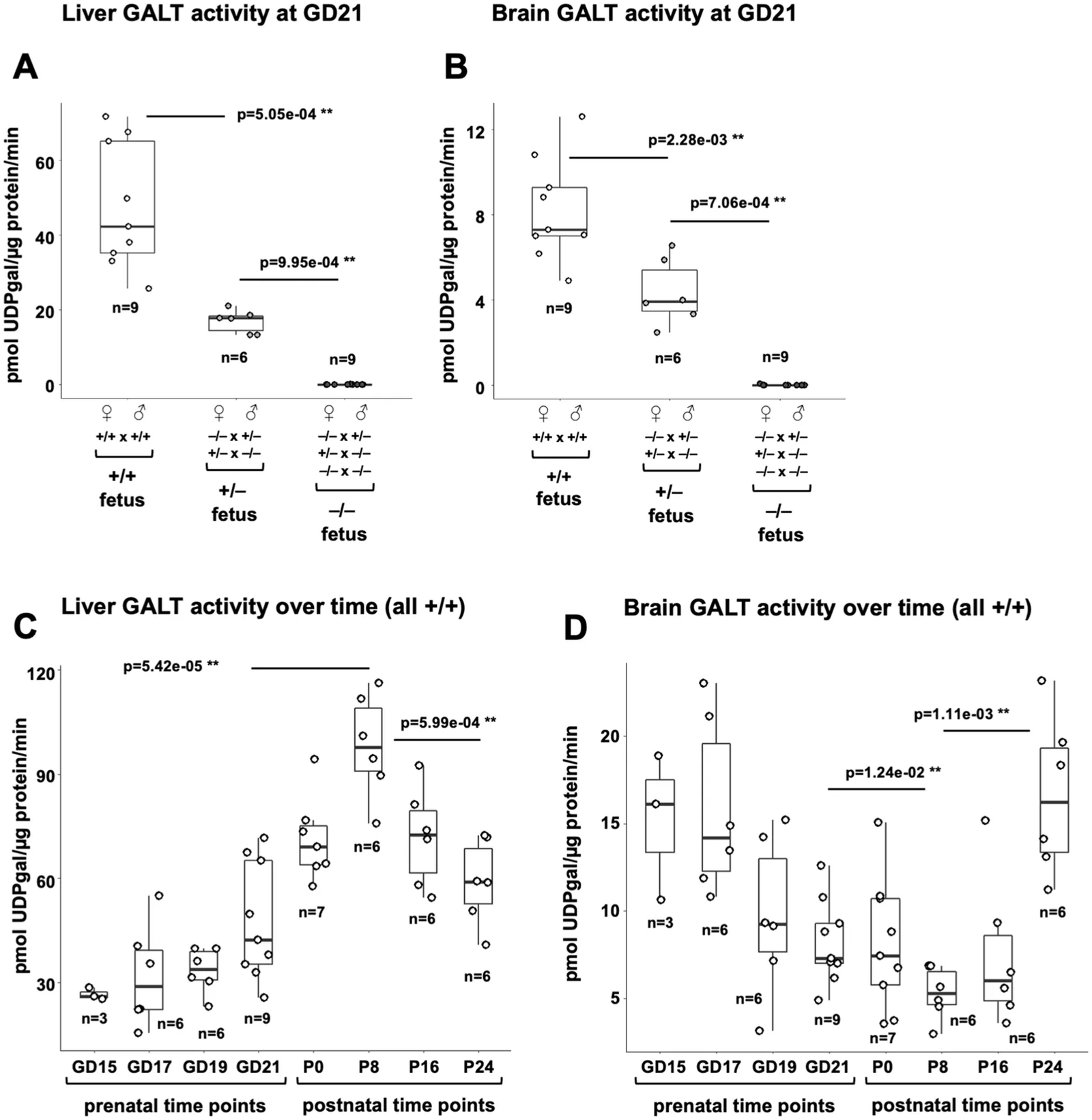
GALT enzyme activity measured in rat fetal liver and brain. **(A)** GALT activity measured in liver samples harvested at GD21 from wild-type, heterozygous, and GALT-null rat fetal pups from the indicated cross types. **(B)** GALT activity measured in brain samples harvested at GD21 from wild-type, heterozygous, and GALT-null rat fetal pups from the indicated cross types. **(C)** GALT activity measured in wild-type rat liver harvested at each of 4 prenatal and 4 postnatal time points, as indicated. **(D)** GALT activity measured in wild-type rat brain harvested at each of 4 prenatal and 4 postnatal time points, as indicated. ** = significant after Bonferroni multiple test correction.

Finally, we explored the possibility of longitudinal changes in GALT activity over time *in utero* and in the weeks after birth. Specifically, we measured GALT activity in samples of liver and brain from wild-type fetal pups at GD15, GD17, GD19, and GD21, and in wild-type postnatal pups at P0 (within 24 hours after birth), P8, P16, and P24 (weaning). Our results confirmed the same basic pattern previously reported in fetal liver, with a transient peak at P8 (see Discussion), but revealed an opposite pattern in brain, where GALT activity was lowest at P8 (Fig 5**, panels C and D**).

## Discussion

The main goal of this study was to test the relationship between abnormal galactose metabolite exposure *in utero* and phenotypic outcome in a GALT-null rat model of CG. Specifically, we leveraged partial metabolic rescue in the maternal-fetal compartment to modify the levels of galactose, galactitol, and gal-1P experienced by GALT-null fetal pups *in utero*. We then asked how each exposure level impacted an accessible measure of fetal outcome – growth.

### Partial metabolic rescue *in utero*

Consistent with prior reports from patients demonstrating elevated galactose metabolite levels in amniotic fluid and fetal tissues [6,7,21,22], all GALT-null fetal pups also showed elevated galactose metabolites in both amniotic fluid and tissues. Further, when a GALT-null fetal pup was developing within a heterozygous mother, the level of amniotic fluid galactitol observed at GD21 was similar to the levels previously reported for second trimester human CG amniotic fluid [21,22]. When the dam was GALT-null, but some pups in the litter were heterozygous, the median amniotic fluid galactitol level was 2.45-fold higher, and when the dam and all fetal pups in the litter were GALT-null, the median amniotic fluid galactitol level was 5.67-fold higher (Fig 2).

Comparing metabolite levels in maternal plasma versus amniotic fluid from GALT-null fetal pups from GALT-null (-/-) x GALT-null (-/-) crosses (Fig 2), we note that the galactose levels were quite similar. However, galactitol levels were much higher in amniotic fluid than in maternal plasma. This pattern may reflect the reality that, late in gestation, amniotic fluid is comprised largely of fetal urine [27], and galactitol is known to concentrate in urine relative to plasma [28].

Whether the metabolic rescue observed among siblings *in utero* was direct, or secondary to impact on the mother’s circulating galactose and galactitol levels, was not clear. Specifically, because the presence of heterozygous fetal pups significantly lowered the galactose and galactitol levels in a GALT-null dam’s plasma, it is possible this change enabled more effective transfer of extracellular metabolites from GALT-null fetal pups to maternal blood at the placenta. Given the absence of strong immediate neighbor effects on the degree of metabolic rescue in GALT-null fetal pups (S6 Fig) supports, but does not confirm, the hypothesis that metabolic rescue among pups may have been secondary to impact on maternal metabolites. It is unlikely the mechanism involved any transfer of actual GALT enzyme or GALT+ cells, since we found no detectable GALT activity in any fetal GALT-null tissues tested – even those from GALT-null pups developing within a heterozygous mother and in the presence of heterozygous siblings (Fig 5**, panels A and B**).

Finally, we noted that partial rescue within the maternal-fetal compartment was clear from studies of all 3 fetal brain metabolites measured, and from fetal liver galactose, but inconsistent from fetal liver galactitol and not at all evident from fetal liver gal-1P (Fig 3). The explanation for this difference is unknown but may reflect different levels of endogenous galactose production in fetal liver versus brain via the action of UDP galactose 4’ epimerase (GALE, see Fig 1). Specifically, if we assume the elevated galactose metabolites detected in GALT-null fetal liver and brain derived from endogenously produced galactose, and if liver produces more galactose than brain, it is possible that galactose is more readily rescued by diffusion at the placenta or across the extra-embryonic membranes, whereas gal-1P, unable to exit the cell, is less susceptible to rescue. If a substantial portion of the galactose metabolized in the brains of these fetal pups originated from liver, it is possible that all galactose metabolites in brain would appear rescued because the liver-derived galactose in fetal circulation was effectively scrubbed at the placenta before reaching the brain.

### GALT activity over time

Previously, Rogers and colleagues [29] reported stable levels of GALT activity in liver samples from wild-type Sprague-Dawley rats *in utero* between about GD15 and birth, and again after birth between about P15 and weaning. Between birth and P15, however, they saw a roughly 3-fold transient spike in GALT activity peaking around P8. Our results confirmed this peak in liver and extended from it documenting a relative trough in GALT activity in brain at approximately the same time. The nearly opposite patterns of change over time in GALT activity observed in post-natal rat liver versus brain are striking and potentially at odds with expectation (Fig 5**, panels C and D**). The implication of these patterns is unclear but may reflect the different sources of galactose typically metabolized in these tissues – dietary galactose in liver and endogenously produced galactose in brain. In a normal rat fetus and pup, the dietary galactose load is negligible *in utero*, increases after birth with nursing, and then decreases again after the pup is weaned. Perhaps endogenously produced galactose in the brain diminishes late in gestation and then increases again as the pup approaches weaning? Prior studies have documented changes over time in whole-body endogenous galactose production in humans, with children producing more galactose than adults [30,31]. We speculate that the level of endogenous galactose production may also vary over time differently in different tissues, resulting in tissue-specific local requirements for GALT.

### Rescue of fetal growth delay

Growth delay of GALT-null fetal pups was not evident until late in gestation (GD21), and then only if the pregnant dam was also GALT-null (Fig 4). Whether the rescue of fetal growth by GALT activity in the dam was secondary to the partial metabolic rescue observed, or resulted from an independent mechanism, is not known. The absence of apparent growth delay among GALT-null fetal pups developing within a heterozygous dam mimics the observation in humans – namely that infants with CG born to carrier mothers show normal birth weight [9].

### Limitations

The GALT-null rat model and experimental design used here had clear strengths, including the ability to alter metabolite levels *in utero*. However, there were also numerous limitations, perhaps the most significant being that the only phenotypic outcome we followed was fetal mass. It is possible GALT-null fetal pups experienced subtle abnormalities in the development of brain or other tissues that we could not detect and that were not rescued. Of course, differences between rats and humans in both prenatal and postnatal development might also confound application of our results presented here to patients. Some of our comparison group cohort sizes were also small, limiting statistical power to see subtle differences.

### Conclusions

The results presented here demonstrate that GALT activity in a pregnant rat dam is sufficient to partially rescue metabolic abnormalities and fully rescue prenatal growth delay in her developing GALT-null fetal pups. This observation offers the possibility that maternal GALT activity might also protect other aspects of prenatal development in GALT-deficiency.

## Materials and methods

### Ethics statement

All animal experiments reported here were approved by the Emory Institutional Animal Care and Use Committee (IACUC) under protocol 201700095. (PI: JL Fridovich-Keil). All post-wean rats in this study had *ad libitum* access to LabDiet 5053 chow and water.

### Rat husbandry and timed pregnancies

As explained in the Introduction and Results, we arranged 3 different types of rat crosses to produce litters that would include GALT-null pups: (i) GALT-null (-/-) females x GALT-null (-/-) males, (ii) GALT-null (-/-) females x heterozygous (+/-) males, and (iii) heterozygous (+/-) females x GALT-null (-/-) males. We also arranged wild-type crosses (+/ + x + /+) to serve as controls. To ensure the accuracy of gestational timing, we used timed pregnancies with males and females housed together for 1 day beginning when the female was in proestrus (see below). Mating was confirmed the next morning by a sperm-positive vaginal smear, and that date was defined as the first day of gestation, GD1. If no sperm were detected in the vaginal smear, the estrus cycle was again monitored until the next proestrus phase, when the female was again paired with a male.

### Monitoring the rat estrus cycle

To determine when each female breeder reached proestrus, we followed a modified version of the wet-stained vaginal smear protocol outlined by Cora et al [32]. Briefly, we collected a daily vaginal cell sample from each rat using 200 μL of sterile 0.9% saline solution drawn up into a pipette fitted with a 200 μL sterile filter tip. The tip was gently inserted into the vaginal opening and flushed 2–3 times. The sample was then transferred to a 1.5 mL microfuge tube and centrifuged at high speed for about 5–10 seconds to pellet the cells. Excess supernatant was discarded from the tube, leaving about 50 μL that was used to resuspend the cell pellet by vortex agitation. 10–20 μL of the resuspended sample was transferred onto a clean glass slide, spread with the side of the pipette tip, and allowed to air-dry at room temperature. Once fully dried, about 200 μL of 0.1% Crystal Violet stain in deionized water was applied to each slide for one minute,  then the slide was rinsed gently with deionized water. Stained slides were visualized under a brightfield microscope. Estrus cycle phase was determined based on the cell composition in the smear, as described previously [32].

### Euthanasia and tissue harvest from pregnant rats

For each cross type, we euthanized 3 pregnant dams representing each of 4 different time points in gestation (GD15, GD17, GD19, and GD21) enabling harvest of plasma from the dam as well as amniotic fluid, liver, and brain from each fetus. As a measure of fetal phenotypic outcome, we also recorded the mass of each fetus at the time of euthanasia. Fetal *GALT* genotypes were confirmed by Transnetyx (https://www.transnetyx.com/) from small pieces of tissue harvested at euthanasia.

Each pregnant dam was anesthetized by isoflurane inhalation until unresponsive, and the chest cavity was then opened to expose the heart. Using a 25G needle fitted onto a 3 mL syringe, we collected 2–3 mL of whole blood directly from the cardiac right atrium and transferred it immediately into a sodium heparin BD Vacutainer tube (366480) which was then inverted 10 times and stored on ice for up to 2 hours until the contents were transferred to 1.5 mL polypropylene tubes for processing. Once the blood sample was collected, the rat was exsanguinated by severing the heart. After five minutes, the fetuses were deemed euthanized by indirect exposure to isoflurane followed by loss of blood flow from the dam.

Following removal of the uterine horn, we numbered the fetuses sequentially from right to left with the fetus closest to the right ovary designated number 1. Each fetus, still in its sac, was carefully removed from the uterine horn and placed into a clean labeled weigh boat on ice. We carefully removed amniotic fluid from each sac using a 25-gauge needle attached to a 1 mL syringe and transferred each sample to a labeled 1.5 mL polypropylene tube on ice. After the amniotic fluid was removed, we carefully opened each sac and separately weighed the fetus and extra-embryonic tissues. The fetal liver and brain were removed and flash-frozen on dry ice and a small tail snip or other fetal tissue sample was collected for genotyping.

#### Sample processing.

Blood and amniotic fluid samples were centrifuged at 2000xg in a microfuge at 4°C for 15 minutes. The amniotic fluid supernatant was aliquoted and snap frozen on dry ice. The plasma layer from each blood sample was also aliquoted and snap frozen. All aliquoted samples were stored at -80°C until further use.

### Euthanasia and tissue harvest from post-natal pups

We harvested post-natal pups at P0, P8, P16, and P24 as described previously [20]. Briefly, pups were anesthetized through isoflurane inhalation as described above for dams. Once unresponsive, pups at P0 and P8 were rapidly decapitated, while pups at P16 and P24 were exsanguinated. We collected liver and brain from each pup; tissues were snap frozen in tubes on dry ice and stored at -80°C until further use.

### Quantifying tissue metabolites and GALT activity

We separated and quantified metabolites extracted from amniotic fluid, plasma, liver, and brain using high performance liquid chromatography (HPLC), as described previously [20]. Specifically, we quantified galactose and galactitol in samples of plasma or amniotic fluid, and galactose, galactitol, and gal-1P in samples of liver or brain.

We quantified GALT enzyme activity in samples of liver and brain also as described previously [20]. Specifically, we analyzed samples from wild-type, heterozygous, and GALT-null fetal pups harvested at GD21, but only from wild-type fetal pups harvested at GD15, GD17, or GD19. We also quantified GALT activity in samples from post-natal wild-type pups harvested at P0 (within 24 hours of birth), P8, P16, and P24.

### Statistical analyses

All statistical analyses presented here were performed using R; graphs presented were also generated using R and R studio [33,34]. P-values presented in all figures were calculated using methods appropriate to the sample sets. Specifically, when all relevant sample sets were normally distributed, we used a parametric test, and when 1 or both sample sets were either not normally distributed, as determined by a Shapiro-Wilk test [35], or were too small to test for normality, we used a non-parametric test. The specific tests applied to calculate specific p-values are presented in S1 Table–S3 Tables. All raw data graphed or analyzed are presented, by figure and panel, in S4 Table–S13 Tables.

## Supporting information

S1 Fig Distribution of *GALT* genotypes among rat fetal pups from homozygote by heterozygote crosses (both directions) harvested at GD15, GD17, GD19, and GD21 (combined). (TIF)

S2 Fig Partial metabolic rescue in rat amniotic fluid at GD15, GD17, and GD19. (A) Galactose levels in amniotic fluid collected at GD15 from GALT-null, heterozygous, and wild-type fetal pups from the indicated cross types. (B) Galactitol levels in amniotic fluid collected at GD15 from GALT-null, heterozygous, and wild-type fetal pups from the indicated cross types. (C) Galactose levels in amniotic fluid collected at GD17 from GALT-null, heterozygous, and wild-type fetal pups from the indicated cross types. (D) Galactitol levels in amniotic fluid collected at GD17 from GALT-null, heterozygous, and wild-type fetal pups from the indicated cross types. (E) Galactose levels in amniotic fluid collected at GD19 from GALT-null, heterozygous, and wild-type fetal pups from the indicated cross types. (F) Galactitol levels in amniotic fluid collected at GD19 from GALT-null, heterozygous, and wild-type fetal pups from the indicated cross types. ** = significant after Bonferroni multiple test correction.
(TIF)

S3 Fig Partial metabolic rescue in rat fetal liver at GD15, GD17, and GD19. (A) Galactose levels in rat liver collected at GD15 from GALT-null, heterozygous, and wild-type fetal pups from the indicated cross types. (B) Galactitol levels in rat liver collected at GD15 from GALT-null, heterozygous, and wild-type fetal pups from the indicated cross types. (C) Gal-1P levels in rat liver collected at GD15 from GALT-null, heterozygous, and wild-type fetal pups from the indicated cross types. (D) Galactose levels in rat liver collected at GD17 from GALT-null, heterozygous, and wild-type fetal pups from the indicated cross types. (E) Galactitol levels in rat liver collected at GD17 from GALT-null, heterozygous, and wild-type fetal pups from the indicated cross types. (F) Gal-1P levels in rat liver collected at GD17 from GALT-null, heterozygous, and wild-type fetal pups from the indicated cross types. (G) Galactose levels in rat liver collected at GD19 from GALT-null, heterozygous, and wild-type fetal pups from the indicated cross types. (H) Galactitol levels in rat liver collected at GD19 from GALT-null, heterozygous, and wild-type fetal pups from the indicated cross types. (I) Gal-1P levels in rat liver collected at GD19 from GALT-null, heterozygous, and wild-type fetal pups from the indicated cross types. * = nominally significant, ** = significant after Bonferroni multiple test correction.
(TIF)

S4 Fig Partial metabolic rescue in rat fetal brain at GD15, GD17, and GD19. (A) Galactose levels in rat brain collected at GD15 from GALT-null, heterozygous, and wild-type fetal pups from the indicated cross types. (B) Galactitol levels in rat brain collected at GD15 from GALT-null, heterozygous, and wild-type fetal pups from the indicated cross types. (C) Gal-1P levels in rat brain collected at GD15 from GALT-null, heterozygous, and wild-type fetal pups from the indicated cross types. (D) Galactose levels in rat brain collected at GD17 from GALT-null, heterozygous, and wild-type fetal pups from the indicated cross types. (E) Galactitol levels in rat brain collected at GD17 from GALT-null, heterozygous, and wild-type fetal pups from the indicated cross types. (F) Gal-1P levels in rat brain collected at GD17 from GALT-null, heterozygous, and wild-type fetal pups from the indicated cross types. (G) Galactose levels in rat brain collected at GD19 from GALT-null, heterozygous, and wild-type fetal pups from the indicated cross types. (H) Galactitol levels in rat brain collected at GD19 from GALT-null, heterozygous, and wild-type fetal pups from the indicated cross types. (I) Gal-1P levels in rat brain collected at GD19 from GALT-null, heterozygous, and wild-type fetal pups from the indicated cross types. * = nominally significant, ** = significant after Bonferroni multiple test correction.
(TIF)

S5 Fig Distribution of metabolites in amniotic fluid, liver, and brain of GALT-null fetal pups from -/- x -/- crosses harvested at GD17, GD19, and GD21. (A) Galactose and galactitol detected in amniotic fluid. (B) Galactose, galactitol, and gal-1P detected in fetal liver. (C) Galactose, galactitol, and gal-1P detected in fetal brain.
(TIF)

S6 Fig Impact of 0, 1, or 2 heterozygous immediate neighbors on metabolite accumulation in GALT-null rat fetal tissues harvested at GD19 and GD21 (combined). (A) Galactose in amniotic fluid. (B) Galactose in fetal liver. (C) Galactose in fetal brain. (D) Galactitol in amniotic fluid. (E) Galactitol in fetal liver. (F) Galactitol in fetal brain. (G) Gal-1P in fetal liver. (H) Gal-1P in fetal brain. * = nominally significant.
(TIF)

S1 Table Statistical tests applied to calculate the p values in Figs 2–5. (DOCX)

S2 Table Statistical tests applied to calculate the p values in S1– S3 Figs.
(DOCX)

S3 Table Statistical tests applied to calculate the p values in S4– S6 Figs.
(DOCX)

S4 Table Raw data presented in Fig 2 panels A-D. Units are as listed in the figure.
(XLSX)

S5 Table Raw data presented in Fig 3 panels A-F. Units are as listed in the figure.
(XLSX)

S6 Table Raw data presented in Fig 4 panels A-B. Units are as listed in the figure.
(XLSX)

S7 Table Raw data presented in Fig 5 panels A-D. Units are as listed in the figure.
(XLSX)

S8 Table Raw data presented in S1 Fig. (XLSX)

S9 Table Raw data presented in S2 Fig panels A-F. Units are as listed in the figure.
(XLSX)

S10 Table Raw data presented in S3 Fig panels A-I. Units are as listed in the figure.
(XLSX)

S11 Table Raw data presented in S4 Fig panels A-I. Units are as listed in the figure.
(XLSX)

S12 Table Raw data presented in S5 Fig panels A-C. Units are as listed in the figure.
(XLSX)

S13 Table Raw data presented in S6 Fig panels A-H. Units are as listed in the figure.
(XLSX)
