# Supplementary material for: Prenatal consequences of GALT deficiency in a rat model of classic galactosemia
Source: PLoS Genet. 2026 Sep 21;22(9):e1012276. doi: 10.1371/journal.pgen.1012276 (PMC13623136; doi:10.1371/journal.pgen.1012276)
Supplement: Supplementary file 1 [file pgen.1012276.s007.docx]

**S1 Table: Statistical tests used to calculate P values in Figs 2-5**

| **Figure (panel)** | **Data sets compared, numbered from left to right in the graph** | **Test applied** |
| --- | --- | --- |
| Fig 2 (A)  GD21 amniotic fluid galactose | set 1 versus set 2  set 1 versus set 3  set 4 versus set 6  set 5 versus set 6 | all Wilcoxon |
| Fig 2 (B)  GD21 amniotic fluid galactitol | set 1 versus set 2  set 1 versus set 3  set 4 versus set 6  set 5 versus set 6 | Wilcoxon  Welch 2 Sample t-test  Wilcoxon  Wilcoxon |
| Fig 2 (C)  Maternal plasma galactose | set 1 versus set 2  set 3 versus set 4 | both Welch 2 Sample t-test |
| Fig 2 (D)  Maternal plasma galactitol | set 1 versus set 2  set 3 versus set 4 | both Welch 2 Sample t-test |
| Fig 3 (A)  GD21 liver galactose | set 1 versus set 2  set 1 versus set 3  set 4 versus set 6  set 5 versus set 6 | all Wilcoxon |
| Fig 3 (B)  GD21 liver galactitol | set 1 versus set 2  set 1 versus set 3  set 4 versus set 6  set 5 versus set 6 | Wilcoxon  Welch 2 Sample t-test  Wilcoxon  Wilcoxon |
| Fig 3 (C)  GD21 liver gal-1P | set 1 versus set 2  set 1 versus set 3  set 4 versus set 6  set 5 versus set 6 | all Wilcoxon |
| Fig 3 (D)  GD21 brain galactose | set 1 versus set 2  set 1 versus set 3  set 4 versus set 6  set 5 versus set 6 | Welch 2 Sample t-test  Welch 2 Sample t-test  Wilcoxon  Wilcoxon |
| Fig 3 (E)  GD21 brain galactitol | set 1 versus set 2  set 1 versus set 3  set 4 versus set 6  set 5 versus set 6 | all Wilcoxon |
| Fig 3 (F)  GD21 brain gal-1P | set 1 versus set 2  set 1 versus set 3  set 4 versus set 6  set 5 versus set 6 | Welch 2 Sample t-test  Wilcoxon  Wilcoxon  Wilcoxon |
| Fig 4 (A)  GD15 fetal mass | set 1 versus set 2  set 1 versus set 3  set 1 versus set 6  set 3 versus set 6 | Wilcoxon  Wilcoxon  Wilcoxon  Welch 2 Sample t-test |
| Fig 4 (B)  GD21 fetal mass | set 1 versus set 2  set 1 versus set 3  set 1 versus set 6  set 3 versus set 6 | Welch 2 Sample t-test  Wilcoxon  Wilcoxon  Wilcoxon |
| Fig 5 (A)  Liver GALT activity by genotype at GD21 | set 1 versus set 2  set 2 versus set 3 | Wilcoxon  Welch 2 Sample t-test |
| Fig 5 (B)  Brain GALT activity by genotype at GD21 | set 1 versus set 2  set 2 versus set 3 | Wilcoxon  Welch 2 Sample t-test |
| Fig 5 (C)  Liver GALT activity over time | set 4 versus set 6  set 6 versus set 8 | both Welch 2 Sample t-test |
| Fig 5 (D)  Brain GALT activity over time | set 4 versus set 6  set 6 versus set 8 | both Welch 2 Sample t-test |
