# Supplementary material for: Prenatal consequences of GALT deficiency in a rat model of classic galactosemia
Source: PLoS Genet. 2026 Sep 21;22(9):e1012276. doi: 10.1371/journal.pgen.1012276 (PMC13623136; doi:10.1371/journal.pgen.1012276)
Supplement: Supplementary file 2 [file pgen.1012276.s008.docx]

**S2 Table: Statistical tests used to calculate P values in S1, S2, and S3 Figs**

| **Supplemental Figure (panel)** | **Data sets compared, numbered from left to right in the graph** | **Test applied** |
| --- | --- | --- |
| S1 Fig (A)  Mendelian ratios of fetal genotypes | set 1  set 2 | both used one-sample exact binomial test |
| S2 Fig (A)  Galactose in amniotic fluid at GD15 | set 1 versus set 2  set 1 versus set 3  set 4 versus set 6  set 5 versus set 6 | all Wilcoxon |
| S2 Fig (B)  Galactitol in amniotic fluid at GD15 | set 1 versus set 2  set 1 versus set 3  set 4 versus set 6  set 5 versus set 6 | all Wilcoxon |
| S2 Fig (C)  Galactose in amniotic fluid at GD17 | set 1 versus set 2  set 1 versus set 3  set 4 versus set 6  set 5 versus set 6 | all Wilcoxon |
| S2 Fig (D)  Galactitol in amniotic fluid at GD17 | set 1 versus set 2  set 1 versus set 3  set 4 versus set 6  set 5 versus set 6 | all Wilcoxon |
| S2 Fig (E)  Galactose in amniotic fluid at GD19 | set 1 versus set 2  set 1 versus set 3  set 4 versus set 6  set 5 versus set 6 | all Wilcoxon |
| S2 Fig (F)  Galactitol in amniotic fluid at GD19 | set 1 versus set 2  set 1 versus set 3  set 4 versus set 6  set 5 versus set 6 | all Wilcoxon |
| S3 Fig (A)  Liver galactose at GD15 | set 1 versus set 2  set 1 versus set 3  set 4 versus set 6  set 5 versus set 6 | all Wilcoxon |
| S3 Fig (B)  Liver galactitol at GD15 | set 1 versus set 2  set 1 versus set 3  set 4 versus set 6  set 5 versus set 6 | Wilcoxon  Wilcoxon  Wilcoxon  Welch 2 Sample t-test |
| S3 Fig (C)  Liver gal-1P at GD15 | set 1 versus set 2  set 1 versus set 3  set 4 versus set 6  set 5 versus set 6 | all Wilcoxon |
| S3 Fig (D)  Liver galactose at GD17 | set 1 versus set 2  set 1 versus set 3  set 4 versus set 6  set 5 versus set 6 | Welch 2 Sample t-test  Welch 2 Sample t-test  Wilcoxon  Wilcoxon |
| S3 Fig (E)  Liver galactitol at GD17 | set 1 versus set 2  set 1 versus set 3  set 4 versus set 6  set 5 versus set 6 | Welch 2 Sample t-test  Wilcoxon  Wilcoxon  Wilcoxon |
| S3 Fig (F)  Liver gal-1P at GD17 | set 1 versus set 2  set 1 versus set 3  set 4 versus set 6  set 5 versus set 6 | all Wilcoxon |
| S3 Fig (G)  Liver galactose at GD19 | set 1 versus set 2  set 1 versus set 3  set 4 versus set 6  set 5 versus set 6 | all Wilcoxon |
| S3 Fig (H)  Liver galactitol at GD19 | set 1 versus set 2  set 1 versus set 3  set 4 versus set 6  set 5 versus set 6 | Welch 2 Sample t-test  Welch 2 Sample t-test  Wilcoxon  Wilcoxon |
| S3 Fig (I)  Liver gal-1P at GD19 | set 1 versus set 2  set 1 versus set 3  set 4 versus set 6  set 5 versus set 6 | Welch 2 Sample t-test  Wilcoxon  Wilcoxon  Wilcoxon |
