# Supplementary material for: Prenatal consequences of GALT deficiency in a rat model of classic galactosemia
Source: PLoS Genet. 2026 Sep 21;22(9):e1012276. doi: 10.1371/journal.pgen.1012276 (PMC13623136; doi:10.1371/journal.pgen.1012276)
Supplement: Supplementary file 3 [file pgen.1012276.s009.docx]

**S3 Table: Statistical tests used to calculate P values in S4, S5, and S6 Figs**

| **Supplemental Figure (panel)** | **Data sets compared, numbered from left to right in the graph** | **Test applied** |
| --- | --- | --- |
| S4 Fig (A)  Brain galactose at GD15 | set 1 versus set 2  set 1 versus set 3  set 4 versus set 6  set 5 versus set 6 | all Wilcoxon |
| S4 Fig (B)  Brain galactitol at GD15 | set 1 versus set 2  set 1 versus set 3  set 4 versus set 6  set 5 versus set 6 | Welch 2 Sample t-test  Welch 2 Sample t-test  Wilcoxon  Wilcoxon |
| S4 Fig (C)  Brain gal-1P at GD15 | set 1 versus set 2  set 1 versus set 3  set 4 versus set 6  set 5 versus set 6 | Welch 2 Sample t-test  Wilcoxon  Wilcoxon  Wilcoxon |
| S4 Fig (D)  Brain galactose at GD17 | set 1 versus set 2  set 1 versus set 3  set 4 versus set 6  set 5 versus set 6 | all Wilcoxon |
| S4 Fig (E)  Brain galactitol at GD17 | set 1 versus set 2  set 1 versus set 3  set 4 versus set 6  set 5 versus set 6 | all Wilcoxon |
| S4 Fig (F)  Brain gal-1P at GD17 | set 1 versus set 2  set 1 versus set 3  set 4 versus set 6  set 5 versus set 6 | all Wilcoxon |
| S4 Fig (G)  Brain galactose at GD19 | set 1 versus set 2  set 1 versus set 3  set 4 versus set 6  set 5 versus set 6 | all Wilcoxon |
| S4 Fig (H)  Brain galactitol at GD19 | set 1 versus set 2  set 1 versus set 3  set 4 versus set 6  set 5 versus set 6 | all Wilcoxon |
| S4 Fig (I)  Brain gal-1P at GD19 | set 1 versus set 2  set 1 versus set 3  set 4 versus set 6  set 5 versus set 6 | Welch 2 Sample t-test  Welch 2 Sample t-test  Wilcoxon  Wilcoxon |
| S5 Fig (A)  Amniotic fluid metabolite proportions at  GD17, GD19, GD21 | all | Kruskal-Wallis |
| S5 Fig (B)  Liver metabolite proportions at  GD17, GD19, GD21 | all | Kruskal-Wallis |
| S5 Fig (C)  Brain metabolite proportions at  GD17, GD19, GD21 | all | Kruskal-Wallis |
| S6 Fig (A)  Amniotic fluid galactose neighbor effects | set 1 versus set 2  set 1 versus set 3  set 2 versus set 3 | all Welch 2 Sample t-test |
| S6 Fig (B)  Liver galactose neighbor effects | set 1 versus set 2  set 1 versus set 3  set 2 versus set 3 | all Welch 2 Sample t-test |
| S6 Fig (C)  Brain galactose neighbor effects | set 1 versus set 2  set 1 versus set 3  set 2 versus set 3 | all Welch 2 Sample t-test |
| S6 Fig (D)  Amniotic fluid galactitol neighbor effects | set 1 versus set 2  set 1 versus set 3  set 2 versus set 3 | all Welch 2 Sample t-test |
| S6 Fig (E)  Liver galactitol neighbor effects | set 1 versus set 2  set 1 versus set 3  set 2 versus set 3 | all Welch 2 Sample t-test |
| S6 Fig (F)  Brain galactitol neighbor effects | set 1 versus set 2  set 1 versus set 3  set 2 versus set 3 | all Welch 2 Sample t-test |
| S6 Fig (G)  Liver gal-1P neighbor effects | set 1 versus set 2  set 1 versus set 3  set 2 versus set 3 | all Welch 2 Sample t-test |
| S6 Fig (H)  Brain gal-1P neighbor effects | set 1 versus set 2  set 1 versus set 3  set 2 versus set 3 | all Welch 2 Sample t-test |
